# Arthroscopic Lesser Trochanter Osteoplasty, Quadratus Femoris Debridement, and Sciatic Neurolysis *via* Posterior Approach for Ischiofemoral Impingement

**DOI:** 10.3389/fsurg.2022.805866

**Published:** 2022-02-16

**Authors:** Qingguo Zhang, Dawei Han, Liwei Ying, Lingchao Ye, Xiangdong Yang, Peihong Liu, Xiaobo Zhou, Tao-Hsin Tung

**Affiliations:** ^1^Department of Sports Medicine, Taizhou Hospital of Zhejiang Province, Wenzhou Medical University, Linhai, China; ^2^Evidence-Based Medicine Center, Public Laboratory, Taizhou Hospital of Zhejiang Province, Wenzhou Medical University, Linhai, China

**Keywords:** ischiofemoral impingement, lesser trochanter, deep gluteal pain, posterior approach, hip arthroscopy

## Abstract

Ischiofemoral impingement (IFI) syndrome is considered the narrowing of the ischiofemoral space (IFS), leading to pathological changes in the quadratus femoris and sciatic nerve, causing posterior hip and sciatica-like pain. Open or arthroscopic resection of the lesser trochanter to enlarge the IFS is the main surgical procedure. However, there is a lack of research on isolated IFI, and currently known surgical procedures are at risk of weakening the flexion strength of the hip joint. In this study, four patients, who were diagnosed with isolated IFI and had undergone arthroscopic treatment with partial resection of the lesser trochanter, debridement of the quadratus femoris, and decompression of the sciatic nerve, were reviewed. To the best of our knowledge, this is the first study to describe the management of IFI using a series of surgical procedures *via* a posterior approach as an effective treatment option. The outcomes of this study broadened the strategies for IFI management.

## Introduction

Ischiofemoral impingement (IFI) syndrome is defined as the narrowing of the ischiofemoral space (IFS) between the lesser trochanter and the ischial tuberosity, causing pathological changes in the quadratus femoris (1). The imaging of IFI indicates lesions within the quadratus femoris, such as edema, tear, and atrophy, and the IFS or quadratus femoris space (QFS) is reduced (2, 3). Long-stride walking (LSW) and IFI tests have been shown to accurately diagnose IFI along with imaging (4). IFI may be related to gender, age, and morphological variation and may also be a manifestation of the hip-spine syndrome (5–7). Patients with IFI usually exhibit chronic posterior hip pain (buttock pain) and sciatica-like pain (non-discogenic sciatica).

Conservative treatments, such as local injection, physical therapy, and non-steroid anti-inflammatory medicines, are usually recommended as initial treatment. If there is no adequate rehabilitative response to first-line therapy, surgical procedures are urgently needed. With the development of arthroscopy technology, arthroscopic resection of the lesser trochanter is considered a reproducible procedure that can achieve improved function and pain relief regardless of an anterior or posterior approach (8–12). Nevertheless, needing to detach the iliopsoas from the lesser trochanter, arthroscopic treatment *via* an anterior approach or *via* a posterior approach for total resection of the lesser trochanter may cause potential complications of weakened hip flexion (8, 9). Hatem described that arthroscopic partial resection of the lesser trochanter *via* a posterior approach could enlarge the IFS and QFS and achieve good functional outcomes (13). However, the study did not exclude patients with concurrent hip disease. To our knowledge, there are few published studies that properly evaluate the results of arthroscopic treatment of isolated IFI. It is hypothesized that arthroscopic partial resection of the lesser trochanter *via* a posterior approach would sufficiently enlarge the IFS and simultaneously debridement of the quadratus femoris and decompression of the sciatic nerve, which would effectively relieve buttock pain in patients with IFI.

This study aimed to evaluate the outcomes of arthroscopic treatment *via* a posterior approach with partial resection of the lesser trochanter and simultaneously debridement of the quadratus femoris and decompression of the sciatic nerve in isolated IFI. This is the first study to describe the management of IFI using a series of surgical procedures *via* a posterior approach for isolated IFI and the outcomes at midterm follow-up. The findings of the present study encourage the use of arthroscopy *via* a posterior approach for the treatment of IFI.

## Materials and Methods

### Diagnosis

This retrospective observational study included patients who were diagnosed with IFI and had undergone arthroscopic treatment *via* a posterior approach between January 2018 and December 2019. Patients with lumbar spinal stenosis (LSS), lumbar disc herniation (LDH), femoral acetabular impingement (FAI), and other peripheral hip impingement diseases were excluded. Ten patients were included in the study. There were two cases of concurrent LDH and LSS, which resulted in lumbar surgery in 1 year. Acetabular arthroplasty and labral repair were performed simultaneously in two patients with FAI. One patient with iliopsoas impingement underwent internal drainage of a cyst and anterior labral repair. Another patient was complicated by a hamstring tear on the side of the ischial tuberosity and tenodesis was performed. Therefore, four out of 10 patients met the criteria.

Ischiofemoral impingement was diagnosed based on comprehensive medical history, symptoms, and physical and imaging examinations. A local injection was also used to identify the cause of pain. Deep gluteal pain, such as buttock and sciatica-like pains, was the main complaint. Positive physical examination findings, such as the LSW test, pain with palpation of the IFS and lesser trochanter, and the IFI test, were considered necessary (4, 13, 14). The LSW test aimed to reproduce impingement. It was defined as positive if the posterior pain was provoked during long stride walking with the affected hip extended, while the pain was alleviated in short-stride walking or hip abduction. Pain with palpation was performed at the IFS in the prone position or contralateral decubitus position and at the lesser trochanter with the affected hip abducted and externally rotated. The IFI test was performed in the lateral decubitus position with passive extension of the affected hip joint. A result was considered positive when the pain was provoked in adduction or in a neutral position and relieved in abduction.

The first choice to identify IFS and QFS was hip MRI in the supine position. Additionally, pathological changes in the quadratus femoris were also considered necessary for IFI diagnosis. IFS was defined as the minimum distance between the ischial tuberosity and the lesser trochanter, the QFS was defined as the minimum space through which the quadratus femoris was passed on axial MRI. The cutoff values of IFS and QFS were set at 17 and 8 mm, respectively. Pathological changes in the quadratus femoris include tearing, edema, and atrophy (2, 3). Other relevant imaging evaluations, such as a standing anteroposterior pelvic radiograph, frog-leg lateral hip radiograph, three-dimensional CT, and lumbar MRI, were also performed to exclude the potential complications that might cause similar symptoms. Ultrasound-guided IFS injection with local anesthetics was also used to identify a focus. Immediate pain relief was considered a positive reaction.

### Surgical Technique

**Figure 1:**
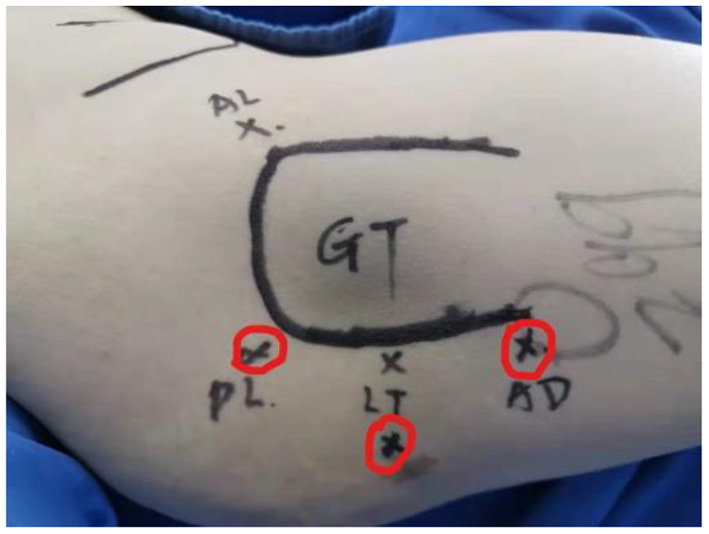
Portal placement for the arthroscopic treatment of IFI. IFI, ischiofemoral impingement. Red cycles mark three portals: posterolateral (PL), lesser trochanter level (LT), and auxiliary distal (AD). GT, greater trochanter.

The surgery was performed in the supine position on a traction table, with internal rotation of the affected hip and without traction. Three portals were usually made: the standard posterolateral portal, mid posterolateral portal, and auxiliary distal posterolateral portal (Figure 1). Arthroscopic cannulas were inserted into the portals. The instruments and arthroscope were introduced through the cannulas and were often switched throughout the procedure to achieve better visualization and convenient operation.

**Figure 2:**
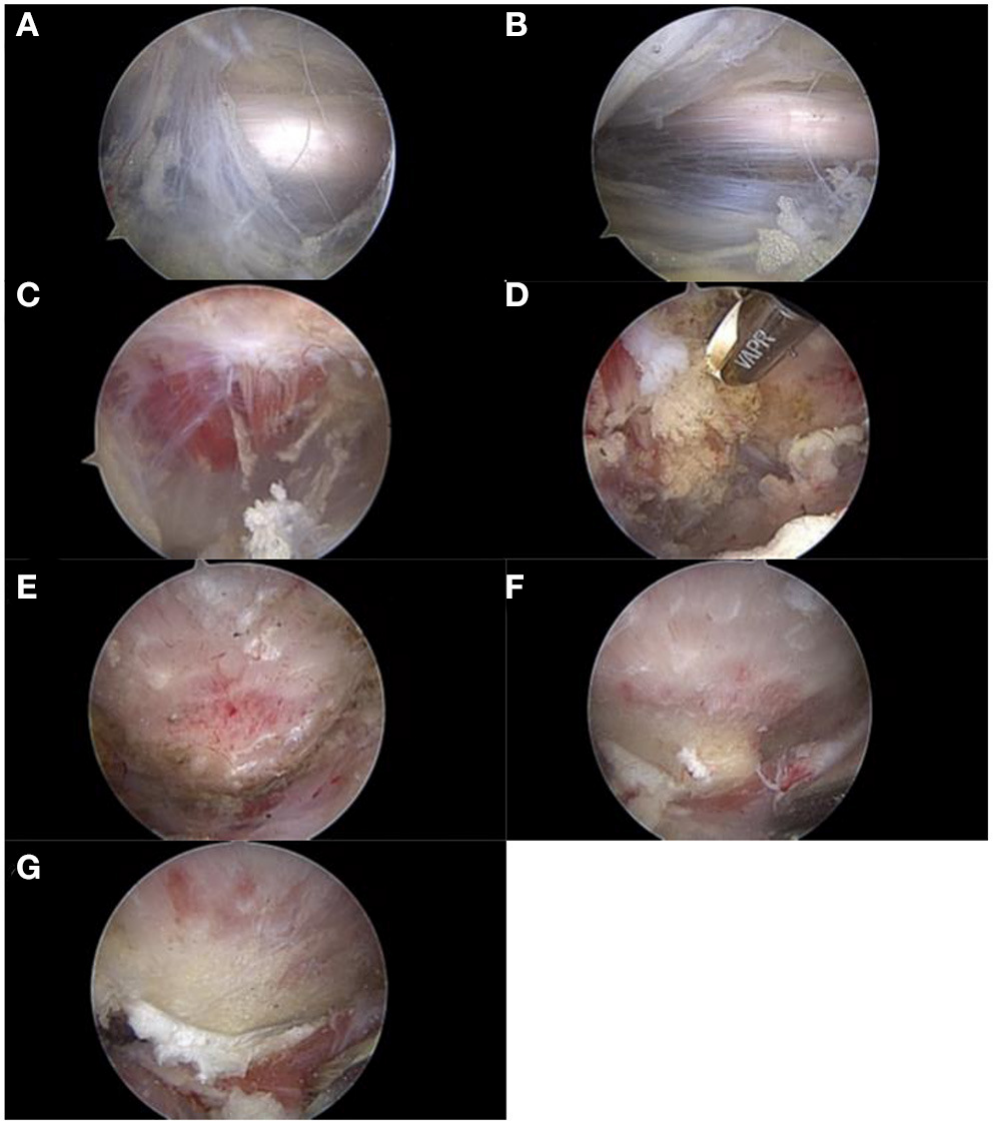
The procedure of arthroscopic treatment for IFI. IFI, ischiofemoral impingement. **(A)** Exposure and evaluation of the sciatic nerve. **(B)** Neurolysis of the sciatic nerve. **(C)** Exposure and evaluation of the quadratus femoris. **(D)** Debridement and fenestration of the quadratus femoris. **(E)** Exposure of the lesser trochanter. **(F)** Reserving part bone to evaluate the depth of excision (1–1.5 burr diameter). **(G)** Posteromedial partial resection of the lesser trochanter.

The course of the sciatic nerve was identified and decompressed if there was an entrapment. The quadratus femoris was then identified and assessed. Edema, tears, and atrophy are usually found. A shaver at low speed and radiofrequency was used for debridement of the quadratus femoris. A window was created in the muscle to allow access to the lesser trochanter. An arthroscopic burr was used to resect the posteromedial one-third of the lesser trochanter to widen the IFS to at least 17 mm and keep the iliopsoas muscle intact. During the operation, the sciatic nerve, proximal circumflex femoral artery, and first distal femoral perforating artery were continuously identified and protected to avoid injury. A probe is usually used for traction (Figure 2).

The distance between the ischium and lesser trochanter was estimated with the hip positioned in extension, flexion, adduction, and rotation to confirm if efficient bone resection and removal of the impingement were achieved. The bone residues needed to be removed to prevent heterotopic ossification, and an anesthetic was injected into the ischiofemoral canal to reduce postoperative pain.

### Postoperative Management

The range of motion of the hip joint was not restricted. Partial weight-bearing as tolerated was recommended with the protection of two crutches for 4–6 weeks. Subsequently, gradual progression to full-weight bearing, single-leg balance training, and abductor strengthening was also initiated. Eight weeks later, recreational sports were allowed. Patients were allowed to start competitive sports at 12 weeks depending on the strength of the abductor muscle group and abdominal muscles.

### Data Collection

**Figure 3:**
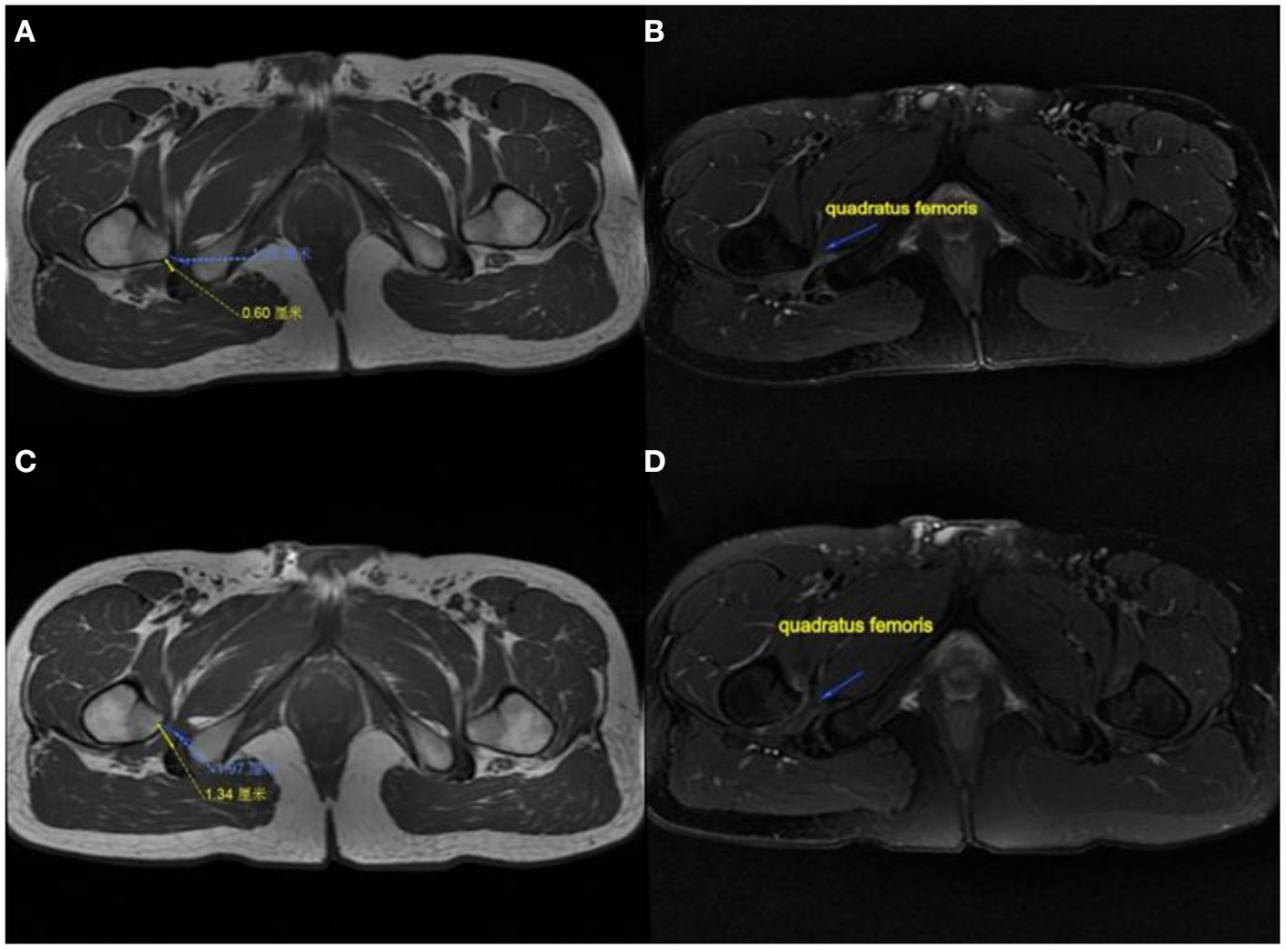
MRI performance of the affected hip in a patient. **(A)** IFS and QFS preoperation show narrowed. **(B)** Quadratus femoris pre-operation showing atrophy and degeneration. **(C)** IFS and QFS at 6 months postoperation show enlargement. **(D)** Quadratus femoris signal returned to normal and increased in volume at 6 months postoperatively. IFS, ischiofemoral space; QFS, quadratus femoris space.

The modified Harris hip score (mHHS) was used to evaluate the function before and after surgery at several follow-up time points. The pain level was scored using a visual analog scale (VAS) from 0 to 10. The Oxford scale was used to assess hip flexion strength from 0 to 5. An MRI of the affected hip was performed 6 months after surgery to observe the IFS, QFS, and the condition of the quadratus femoris (Figure 3). The clinical results were evaluated using IFI and LSW tests (Table 1). Postoperative complications were recorded in the medical records during follow-up.

**Table 1:** Summary of clinical features, imaging findings, and outcomes of surgery of four patients with IFI.

	**Patient 1**	**Patient 2**	**Patient 3**	**Patient 4**	**Mean**
Age, yr	29	32	48	51	40
Gender	M	M	F	F	
Duration of symptoms until surgery, mo	8	10	18	12	12
Major complaint	Buttock and sciatica-like pain	Buttock and sciatica-like pain	Buttock and sciatica-like pain	Buttock and sciatica-like pain	
Posterior pain with long-stride gait (long stride walking test)	Pos	Pos	Pos	Pos	
Pain with short-stride gait	Neg	Neg	Neg	Neg	
IFI test	Pos	Pos	Pos	Pos	
Flexion-adduction-internal rotation test	Neg	Neg	Neg	Neg	
Dynamic internal/external rotatory impingement test IFS on axial MRI, mm	Neg	Neg	Neg	Neg	
Preoperative	16.3	14.8	12.3	10.2	
Postoperative	26.5	23.7	26.3	24.0	
QFS, mm Preoperative	*5.7*	5.1	4.9	4.5	
Postoperative	*15.6*	14.6	14.3	13.9	
Length of follow-up, mo	*24*	18	24	30	
Time to return to sport, mo	*3*	3	3	3	
mHHS, points Preoperative	*61*	61	51	51	
Final follow-up	*100*	100	100	100	
VAS score for pain Preoperative	*4*	6	6	6	
Final follow-up	*0*	0	0	2(over exercise)	

*F, female; M, male; mHHS, modified Harris hip score; Neg, negative; Pos, positive. IFS, ischiofemoral space; QFS, quadratus femoris space*.

### Statistical Analysis

Statistical analyses were performed using SPSS 22.0. Basic data characteristics and distribution of the effective samples were clarified using descriptive statistical analysis. A repeated-measures ANOVA was performed to clarify the bivariate analysis results of the mHHS. Statistical significance was set at *p* < 0.05.

## Results

**Figure 4:**
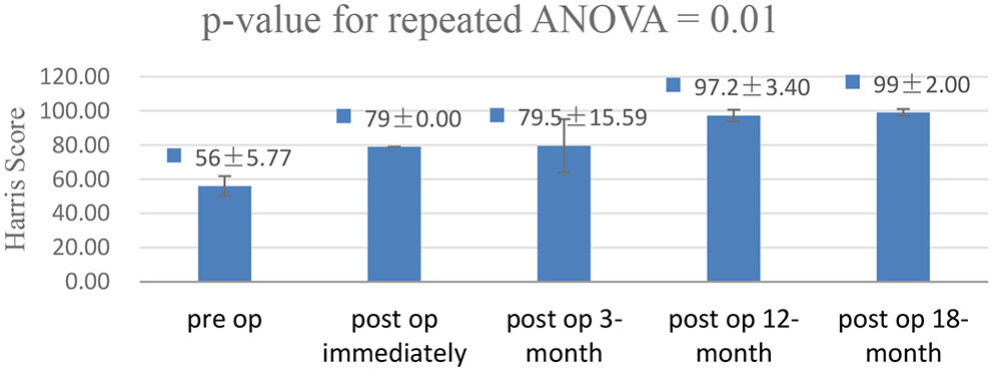
A repeated-measures analysis of variance was performed to clarify the bivariate analysis results of the modified hip Harris score (mHHS). The mHHS showed significant improvement in the functional outcome (*p* = 0.01).

Four out of 10 patients (two men and two women) met the criteria, and the average age was 40 years (range, 29–51 years). All four patients complained of buttock pain and sciatica-like pain when performing locomotor activities before surgery, especially when walking fast and taking long strides. The average duration of symptoms was 12 months (range, 8–18 months). The IFI and LSW tests were positive in all four patients, whereas the hip impingement and internal and external rotatory impingement tests were negative. The mean IFS was 13.4 mm (range, 10.2–16.3 mm) and the mean QFS was 5.1 mm (range, 4.5–5.7 mm) in four patients before surgery, according to MRI. The average follow-up period was 24 months (range, 18–30 months). The mean IFS was 25.1 mm (range, 23.7–26.5 mm) and the mean QFS was 14.6 mm (range, 13.9–15.6 mm) in all patients after surgery. The mHHS showed significant improvement (*p* = 0.01; Figure 4), and the mean elevation was improved by approximately 43 points (range, 39–49 points). The mean VAS score decreased from 5 (range, 4–6) before surgery to 0.5 (range, 0–2) at the final follow-up. All four patients recovered their exercise ability after 3 months. Among them, three patients returned to the same level as before at 6 months after surgery (Table 1).

**Figure 5:**
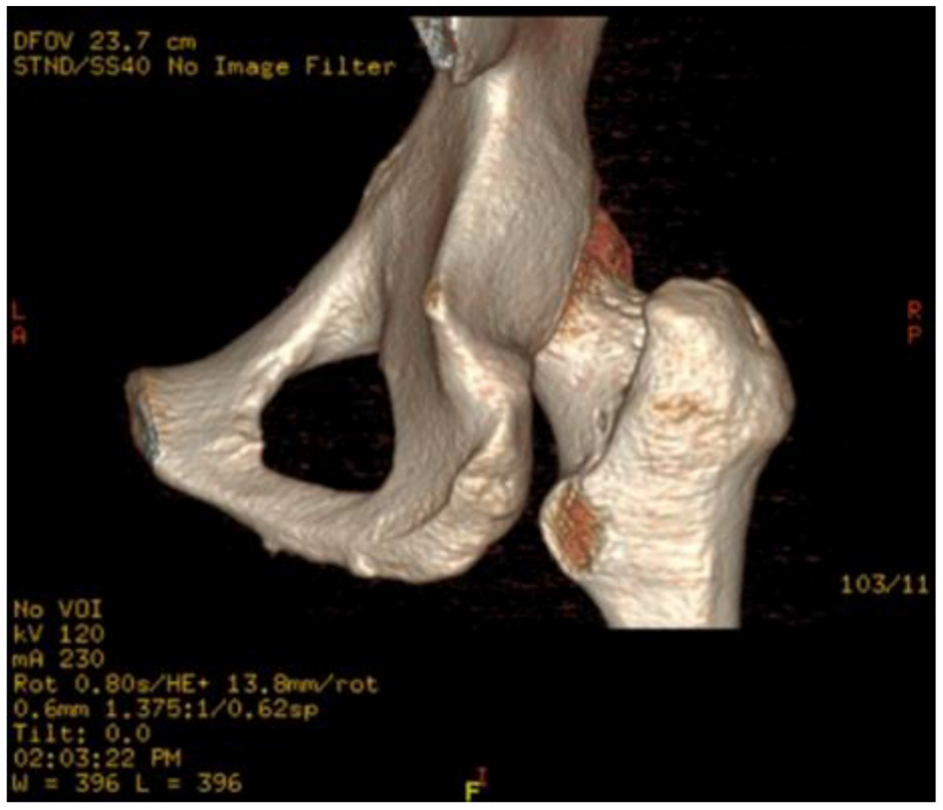
Postoperative three-dimensional CT of the affected hip. Approximately one-third of the posterior medial lesser trochanter was resected.

After surgery, no sciatic or vascular complications were observed. Excluding pain, flexion weakness of the hip was not observed at follow-up. Heterotopic ossification or femoral head necrosis signs were not observed on postoperative radiographs and MRI. Partial resection of the posteromedial one-third of the lesser trochanter was observed on postoperative three-dimensional CT (Figure 5). The edema of the quadratus femoris was vanished on postoperative MRI (Figure 3).

## Discussion

With the development of arthroscopy, arthroscopic resection of the lesser trochanter is considered less invasive and efficient for the treatment of IFI. Some researchers have reported arthroscopic partial or complete resection of the lesser trochanter *via* an anterior approach (9–12). However, to visualize the lesser trochanter, detachment of the iliopsoas tendon is required, which may result in flexion weakness of the hip (9). Gomez-Hoyos et al. (15) described the footprint location of the iliopsoas tendon on the lesser trochanter, demonstrating that the iliopsoas tendon insertion was consistently located at the top of the anterior wall. Therefore, total resection or partial resection of >50% of the tip *via* a posterior approach will also have the risk of damaging the iliopsoas insertion (8, 15). Rafael described an endoscopic procedure in which the detached iliopsoas tendon was re-fixed to avoid the risk of hip flexion weakness and anterior hip instability (16). However, functional results from further investigations are lacking. Furthermore, the procedure theoretically increases the difficulty of surgery and recovery time. This study demonstrated that arthroscopic posteromedial partial resection of the lesser trochanter and management of the surrounding pathological tissue *via* the posterior approach is an effective strategy for IFI.

According to Torriani, IFS was measured from the lateral cortex of the ischial tuberosity to the posteromedial cortex of the lesser trochanter, and the QFS was measured from the posteromedial surface of the lesser trochanter to the superolateral surface of the hamstring tendons (2). Both spaces were defined as the smallest distances on axial MRI. Vicentini (17) and Kivlan (18) demonstrated that IFS and QFS changed with hip rotation. The lesser trochanter is close to the ischium during the external rotation of the femur and moves away during the internal rotation of the femur. The dynamic trajectory of the lesser trochanter showed a posteromedial crescent shape. Theoretically, this means that the posteromedial margin of the lesser trochanter is the closest part to the ischial tuberosity and the main part contacts the quadratus femoris throughout the entire process. Consequently, posteromedial partial resection of the lesser trochanter may be the most efficient method to enlarge the space and eliminate abnormal contact. Moreover, one-third of partial posteromedial resection causes minimal damage to the iliopsoas tendon insertion, which facilitates flexion strength of the affected hip and decreases the risks of potential stress fracture and intra-abdominal fluid extravasation (8, 19). In our study, postoperative IFS and QFS exceeded the cutoff points in all four cases, and flexion weakness of the hip was not found. Hatem (13) described the surgical procedure with partial resection *via* a posterior approach for the treatment of five patients with IFI. Sufficient IFS and QFS and satisfactory functional results were obtained, which is in agreement with our study. However, the research did not exclude cases with FAI, and concomitant intra-articular abnormalities were treated simultaneously, which might have influenced the clinical results.

Although IFI is anatomically defined as a decrease in IFS and QFS, there may be cases in which the space is normal in MRI imaging, but impingement is present. In addition, some patients with significantly reduced IFS do not show symptoms of IFI (8, 17, 20, 21). In our study, all four patients presented with unilateral symptoms of deep gluteal pain and sciatica-like pain, with significantly reduced IFS and QFS in the bilateral hip joint. Considering that the quadratus femoris is the most affected structure, pathological changes in the quadratus femoris (edema, degeneration, and tear of the quadratus femoris) may be the most direct cause of gluteal pain. Consequently, debridement of the quadratus femoris is essential and effective for pain relief. The sciatic nerve crossed behind and adhered to the quadratus femoris at 4 mm from the femoral border. The reduced IFS induces entrapment of the sciatic nerve, caused by the formation of fibrovascular bands due to a mechanical conflict between the ischium and the lesser trochanter (22, 23). In addition, lesions of the quadratus femoris can lead to an inflammatory response that stimulates the sciatic nerve and causes sciatica-like pain. Therefore, it is important to perform sciatic nerve identification and exploration during the surgical procedure, and neurolysis is needed if there is nerve entrapment (8, 24). Although the posterior approach has the potential to cause iatrogenic injury of the sciatic nerve and surrounding arteries, as described by some authors (23, 25), no cases have been reported in the literature. Moreover, it is convenient to explore and identify the sciatic nerve and artery *via* a posterior approach, and it is less likely to cause damage than the anterior approach with the gentle operation. Furthermore, using a probe to pull and obstruct the nerve and artery *via* an auxiliary approach can help reduce the risk. In this study, there was no evidence of damage to important arteries or the sciatic nerve. Moreover, the posterior approach allows simultaneous handling of other subgluteal pathologies, if necessary (25, 26).

To our knowledge, the mechanism underlying the initial onset of IFI is unclear. According to studies (5–7), IFI may be related to gender, age, and morphological variation and may also be a manifestation of the hip lumbar syndrome. Similar to the treatment of subacromial impingement and FAI, osteogenesis, debridement, and neurolysis can also relieve the symptoms; however, the treatment is only targeted at the disease outcomes. The findings of the present study encourage the use of arthroscopy *via* a posterior approach for the treatment of IFI. However, surgery is the only available treatment option. To better diagnose and treat IFI, further studies on the etiology, pathogenesis, and the development of a holistic treatment plan are necessary.

This study has some limitations. First, the sample size was too small with only four patients. IFI is not a frequent pathology, and we excluded cases complicated by other diseases that might cause similar symptoms, such as LDH, LSS, and FAI. During the surgery, simultaneous treatment for other intra- and extra-articular pathologies was not performed, which may minimize the interference with the conclusion. Second, the results of functional and imaging assessments are only from the mid-term follow-ups, and further studies on long-term follow-up results are required. Third, functional assessment of mHHS in patients without the hip disease has inherent restrictions. Finally, for technical reasons, it is difficult to measure the lesser trochanter version, which may be retroverted in patients with IFI. Further imaging studies are needed to better understand the pathogenesis of IFI and develop reasonable treatments.

## Conclusion

This study suggests that arthroscopic treatment with partial resection of the lesser trochanter and simultaneous management of the surrounding pathological tissue *via* the posterior approach is an effective treatment option for IFI. Our posterior approach may provide a promising treatment option in cases of failure of conservative management of IFI.

## Data Availability Statement

The original contributions presented in the study are included in the article/supplementary material, further inquiries can be directed to the corresponding authors.

## Ethics Statement

The studies involving human participants were reviewed and approved by Ethics Committee of Taizhou Hospital of Zhejiang Province Affiliated to Wenzhou Medical University. The patients/participants provided their written informed consent to participate in this study. Written informed consent was obtained from the individual(s) for the publication of any potentially identifiable images or data included in this article.

## Author Contributions

QZ, PL, and XZ: conceptualization and draft preparation. QZ: revision. DH, LY, and XY: data collecting. DH, LY, XY, and T-HT: data analysis. PL and XZ: supervision. LY: writing original draft and revision. T-HT: formal analysis. All authors contributed to the article and approved the submitted version.

## Conflict of Interest

The authors declare that the research was conducted in the absence of any commercial or financial relationships that could be construed as a potential conflict of interest.

## Publisher's Note

All claims expressed in this article are solely those of the authors and do not necessarily represent those of their affiliated organizations, or those of the publisher, the editors and the reviewers. Any product that may be evaluated in this article, or claim that may be made by its manufacturer, is not guaranteed or endorsed by the publisher.
